# Breakthrough in 4π ion emission mechanism understanding in plasma focus devices

**DOI:** 10.1038/srep38843

**Published:** 2016-12-12

**Authors:** Mehdi Sohrabi, Arefe Zarinshad, Morteza Habibi

**Affiliations:** 1 Department of Energy Engineering and Physics, Amirkabir University of Technology, Tehran, Iran

## Abstract

Ion emission angular distribution mechanisms in plasma focus devices (PFD) have not yet been well developed and understood being due to the lack of an efficient wide-angle ion distribution image detection system to characterize a PFD space in detail. Present belief is that the acceleration of ions points from “anode top” upwards in forward direction within a small solid angle. A breakthrough is reported in this study, by mega-size position-sensitive polycarbonate ion image detection systems invented, on discovery of 4π ion emission from the “anode top” in a PFD space after plasma pinch instability and radial run-away of ions from the “anode cathodes array” during axial acceleration of plasma sheaths before the radial phase. These two ion emission source mechanisms behave respectively as a “Point Ion Source” and a “Line Ion Source” forming “Ion Cathode Shadows” on mega-size detectors. We believe that the inventions and discoveries made here will open new horizons for advanced ion emission studies towards better mechanisms understanding and in particular will promote efficient applications of PFDs in medicine, science and technology.

A plasma focus device (PFD) is a strong multidisciplinary source of electrons, soft and hard x-rays, ions and neutrons (with deuterium gas) of interest for different applications^1^,^2^,^3^,^4^,^5^. Some applications include radioisotope production^6^,^7^, thin film material processing^8^, synthesis of nanophase materials^9^,^10^, ion implantation^11^, lithography^12^, radiography imaging^13^ and neutron sources^14^.

In PFD space, ion measurements are made to characterize beams of ions in terms of ion energy and angular fluence distributions for mechanisms understanding and to promote applications. In such line of studies, the emission of ions in conic geometry within a small solid angle is confirmed by measuring some ion characteristics only at certain locations in a PFD space, usually along the X or Y axes, above the vacuum chamber. Some researchers believe that a PFD generates ions during the progress of sausage instability towards the top of a PFD chamber in a conical fashion within a small solid angle with ion energies ranging from tens of keV to few MeV^15^,^16^,^17^. As a result, for all plasma-material interaction studies by means of different scale PFDs from PF-1000^14^,^18^,^19^ to miniature PFDs^20^, the specimens are usually placed along the z-axis of the PFD chamber to be exposed to ions by applying several pinch shots^21^. Many exclusive studies have been performed on ion emission angular distribution profiles on X or Y axes of PFDs; some studies have reported maxima^22^,^23^,^24^,^25^ and some have shown minima^26^,^27^,^28^,^29^,^30^,^31^, on the profiles, on or around the z-axis. Some theoretical models have also been developed to justify ion angular distribution profiles around the z axis^22^,^23^,^24^,^29^,^30^,^32^,^33^,^34^,^35^.

Some studies have focused on wider-angle ion emission distributions in PFDs^7^,^18^,^35^,^36^,^37^,^38^. Measurement of high energy neutrons at an angle of about 180° assumed to be due to energetic ions in the backward direction of the electrode axis^36^; detection of deuterium accelerated in the backward direction more than 90° in a PFD by using activation of graphite foil^7^; measurement of deuterium at 0°, 60° and 180° angular positions in the upstream and downstream directions along the *z*-axis of a PF-1000 device by PM-355 detectors suggesting that a portion of fast ion population are originated from the regions between electrode arrays^18^; ion images recorded during discharges with a complex structure, usually in the form of a central bunch and an annular stream composed of many micro-beams^37^; development of a full kinetic model proposed on predicting ion beams in both z directions, albeit with much stronger beams in the forward direction^35^, and last but not least the observation of a wide-angle x-ray image formation with cathode x-ray shadows in a PFD space^39^. In particular, a recent study on hydrogen ion angular distributions in 19 kJ PFD using CR-39 detectors displayed at 9 discrete angles from −80° to 90° on a circular frame of 11 cm diameter around the anode tip concluded that high current density ion beams are observed even in far off-axis positions and the ions with energies lower than 300 keV are distributed widely over the emission angle of ±80°, while the higher-energy protons (>1 MeV) are confined in the half cone angle of ±30° 38.

The conflicting results reported so far on PFD ion angular distribution studies while are mostly true facts observed in a PFD space, they can be considered as special cases of a broader 4π ion emission angular distributions in PFD space, as observed in our detailed studies. The main reasons for such conflicting data and understanding are due to the fact that the mechanisms through which ions are emitted and distributed in a PFD space have not yet been well developed, demonstrated and understood, albeit extensive experimental studies made and models proposed^1^,^3^,^24^,^34^,^40^. However, most experimental studies on ion fluence and energy measurements made so far are by using one or a short array of single small detectors on the z-axis or within a small solid angle on or around it or on X or Y axes by applying several pinch shots. The detectors usually used in such studies include activation detectors^7^,^41^, magnetic analyzers^42^, Faraday cups (FC)^25^,^28^,^43^, Thomson spectrometer^44^ and in particular polymer track detectors like CR-39 (polyallyl-diglycol-carbonate film), PM-355 (super-grade polyallyl-diglycol-carbonate film), CN (cellulose nitrate film) and LR-115 (red-dyed cellulose nitrate film). These polymer detectors can register tracks of ions such as protons, deuterons, alpha particles or heavier ions usually by a chemical etching process followed by microscopic observation and counting^7^,^16^,^18^,^27^,^45^.

The rationale for the limitations in observing ion emission angular distributions in a wider-angle PFD space and in turn better mechanism understanding has been the lack of an ion angular distribution image detection system with a potential to provide wide-angle position-sensitive matrix data under single pinch shots. Recently, efficient continuous position-sensitive polycarbonate (PC) detectors (a polymer track detector) processed by electrochemical etching (ECE) methods have been successfully applied over the “anode top” for helium, nitrogen and neon ion angular distribution image profile studies^46^,^47^,^48^. In particular, by applying mega-size PC (MSPC) detectors around the inner PFD wall, it was discovered that the ions not only are emitted and distributed upwards, downwards and in all directions over 4π PFD space after plasma pinch instability, ions are also escaped radially during the axial acceleration of the plasma sheath before the axial phase. As a result, a comprehensive mechanism containing ion acceleration in 4π PFD space and ion radial run-away during the axial phase required extensive studies. Accordingly, a “4π ion emission hypothesis” was developed in this study with the incentive to be further studied and verified in PFD space. In order to characterize this “hypothesis” by real wide-angle measurements and analysis in 4π PFD space, it was the purpose of this study to:

1. develop and apply position-sensitive MSPC ion image detectors processed by mega-size ECE (MS-ECE) chamber systems for 4π ion emission angular distribution image studies in a PFD space,
2. study and verify sources of ion emissions from the “anode top” and the “anode electrodes array” in 4π PFD space, and
3. explore other relevant phenomena with an aim to enhance better understanding of the mechanisms involved and last but not least to promote efficient advanced applications of PFD space in medicine, science and technology.

## Results

### Ion emission angular distribution image detection

Position-sensitive MSPC ion image detectors (250 μm thick) processed in MS-ECE chamber systems, as discussed in the method section below, were applied to study 4π ion emission angular distributions in 3.5 kJ Mather-type PFD space^49^. Helium gas was applied as the main gas in this study although other gases such as hydrogen, deuterium, nitrogen and neon were also used in our parallel studies. Two main MSPC detectors were used; single circular MSPC detectors (26 cm in diameter, the size of the PFD cylinder base) to develop ion emission angular distribution images at known distances from the “anode top” and single rectangular MSPC detectors (38 cm × 81 cm) to develop ion angular distribution image on the PFD wall. The two single MSPC detectors were simultaneously installed in the PFD inner wall areas and exposed to helium ions at a 7.5 mbar pressure under only a single pinch shot. The experiments were repeated by placing each time a circular MSPC detector perpendicular to the anode axis at either 6, 9, 12, 15, 18 or 21 cm distances from the “anode top”, while a wall MSPC detector was placed and exposed together with a circular detector when placed either at 6, 15 or 21 cm distances from the “anode top”. Also, in order to verify the role of the “anode top” and the “anode cathodes array” on the radial run-away ions, smaller rectangular MSPC detectors (21 cm × 50 cm) were placed around the cathodes; either one wrapped around and attached to the cathodes or installed around the cathodes with 3 cm and 7.5 cm distances respectively away from the cathodes and anode centers. Further, in order to verify if any ions reach the PFD ground cylinder base, additional small 3 cm × 3 cm PC detectors were laid on the ground cylinder base between the anode and cathodes as well as between the cathodes and the PFD inner wall. After exposure to helium ions, the detectors were processed either in MS-ECE chambers or in small ECE chambers at optimized ECE conditions depending on the detector size, as discussed in the method section (Fig. 7).

**Figure 1:**
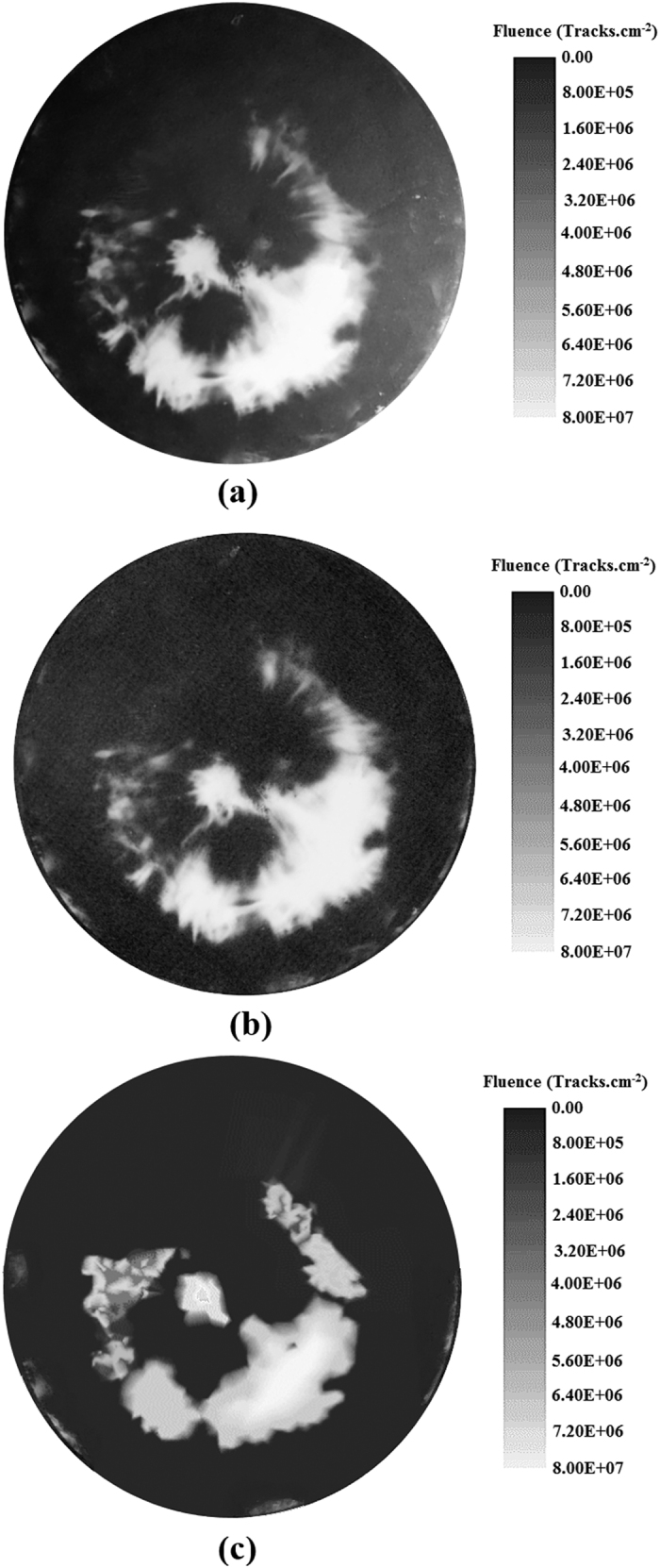
(**a–c**) Images of ion distributions on a circular MSPC detector. Images of helium ion track distributions on an ECE-processed circular MSPC detector (26 cm diameter) when placed at 6 cm distance from the “anode top” as observed by the unaided eyes and also developed by direct photography (**a**), scanned by a normal scanner (**b**), and plotted as ion isofluence distribution using Origin Software (**c**).

Depending on the purpose of each experiment, the circular and rectangular MSPC detectors as well as small PC detectors, as arranged in the PFD space (Fig. 7), were exposed to helium ions under a single pinch shot. After the ECE processing of the detectors, 4π ion emission distribution images were well registered on the detectors, diagnosed and analyzed. The images can be directly observed and analyzed by the unaided eyes, photographed by a digital camera, scanned by a normal scanner, and plotted as ion isofluence or isoenergy distributions by Origin Software. They are based on track densities determined by counting the ion tracks under an ordinary microscope, as shown for example on a circular MSPC detector exposed at 6 cm from the “anode top” (Fig. 1a,b,c).

**Figure 2:**
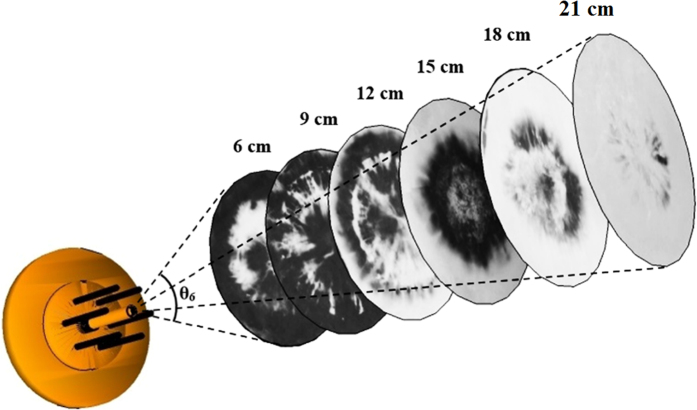
Photographs of helium ion distribution images on 6 circular MSPC detectors. Images of helium ion emission angular distributions on 6 circular MSPC detectors each placed at either 6, 9, 12, 15, 18 or 21 cm distances from the “anode top” respectively and exposed at a time to a single pinch shot.

The formation of an ion angular distribution image on either a circular or a rectangular MSPC detector exposed in PFD space under a single shot depends, among other parameters, on the distance of the circular MSPC detector from the “anode top”. In this context, images of ion emission angular distributions on 6 circular MSPC detectors, each placed at either 6, 9, 12, 15, 18 or 21 cm distance from the “anode top” and exposed at a time to ions at a single pinch shot at a helium gas pressure of 7.5 mbar are shown in Fig. 2.

As can be seen in Fig. 2, the ion angular distribution image on each circular MSPC detector exposed at a known distance from the “anode top” is quite different from those of others, which are discussed further in this paper.

### Ion isofluence and isoenergy distributions

Development of isofluence and isoenergy distributions of ions in PFD space simplifies identifying locations in a PFD with equal ion fluence and ion energy in particular on the inner walls where specimens can be exposed. These types of distributions also specify ion hot spots and in general, the ion field uniformity for exposing large number of samples at the same fluence and energy conditions in PFD space. The MSPC image detector system provides such a possibility for developing efficient isofluence and isoenergy over large inner areas of a PFD. In this context, ion track density and track diameters of positions in MSPC detectors were determined under a light microscope. Due to difficulty in determination of ion track density and diameters of tracks in particular at high track densities, a new “paired-detector comparative method”, recently developed in our laboratory (yet to be published), and was applied. In this method, a microscopic image of helium ion tracks on an area of a MSPC detector exposed to ions with unknown fluence and energy is compared with that of a microscopic image of alpha particle tracks in a template PC detector with a known fluence and energy. The template detectors have been precisely calibrated by exposing each to alpha particles of known fluence and energy by degrading ~5.49 MeV alpha particles of a well collimated ^241^Am source in air at difference distances from the source.

**Figure 3:**
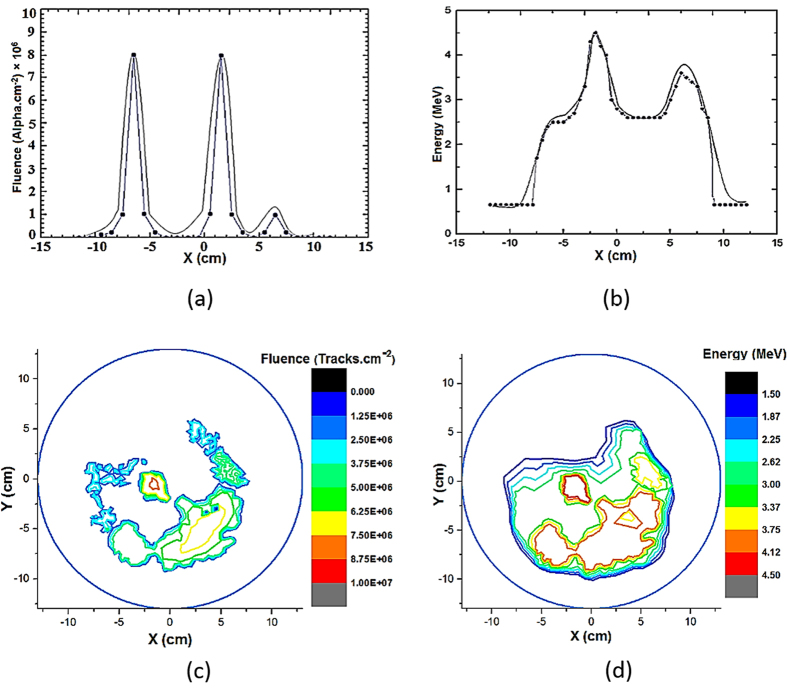
(**a–d**) Ion angular isofluence and isoenergy distributions. Helium ion fluence (**a**) and ion energy (**b**) angular distributions profiles on the X axis as well as helium ion isofluence (**c**) and isoenergy (**d**) distributions on a circular MSPC detector (26 cm diameter) exposed at a distance of 6 cm from the “anode top” (see also Figs 1 and 2).

In most PFD studies, 2D ion angular distributions have been developed within a relatively small solid angle above or around the z axis over the “anode top” on the Y or X axis. Such studies usually report the position of maxima or minima on the X or Y axes profiles^22^,^23^,^24^,^25^,^26^,^27^,^28^,^29^,^30^,^31^. As an example of 2D ion angular distribution profiles, Fig. 3(a,b,c,d) shows helium ion fluence (a) and ion energy (b) angular distribution profiles along the X axis of the PFD as well as 2D isofluence (c) and isoenergy (d) distributions on a circular detector exposed at a distance of 6 cm from the “anode top”.

The general trend of the 2D ion fluence angular distribution profiles on a circular MSPC detector exposed at a distance 6 cm (Fig. 3a) and at other distances from the “anode top” is similar to the trend of profiles reported on some other ion angular distribution profiles, using a single or an array of few detectors, on which some minima or maxima have been reported^22^,^23^,^24^,^25^,^26^,^27^,^28^,^29^,^30^,^31^.

As can be seen in Fig. 2, the images on the circular MSPC detectors and in turn the 2D angular distribution profiles (Fig. 3a) depend on the distance of the circular detector from the “anode top” and the diameter of the circular detector on which the profile is determined. Some other parameters such as type of PFD, type of the detector, number of pinch shots, type of ions and gas pressure also affect such angular distribution profiles. A circular detector has infinite diameters, each can be considered as a PFD axis, resulting to infinite type of angular distribution profiles. Therefore, each ion emission angular distribution profile reported by using different types of detectors and PFDs^22^,^23^,^24^,^25^,^26^,^27^,^28^,^29^,^30^,^31^, can be considered only as specific cases of full angular distributions image observed on a circular MSPC detector (Fig. 2).

The MSPC detectors being position sensitive also can provide matrix information on isofluence and isoenergy distributions (Fig. 3c,d). These distributions were based on determination of the ion fluence and energy on each position of for example a circular MSPC detector using the “paired-detector comparative method” as discussed above. By such investigations, helium ion energies were estimated to extend from ~0.3 to ~4.5 MeV, as also reported before^48^.

### Ion 3D emission angular distributions in 4π PFD space

**Figure 4:**
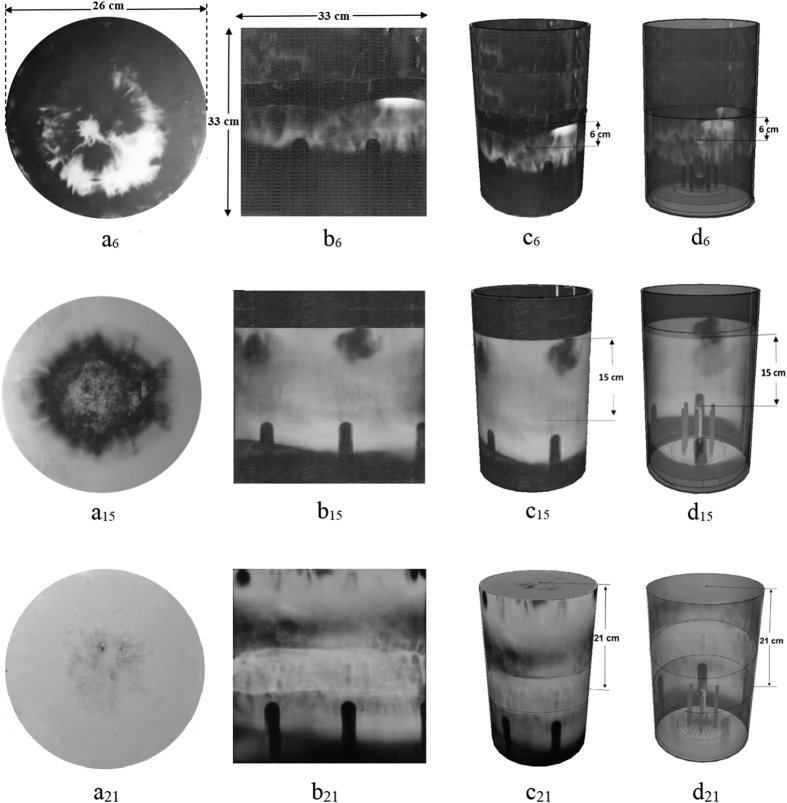
(**a–d**) 3D ion emission distribution images in 4π PFD space. (a_6,15,21_) Images of helium ion angular distributions on circular MSPC detectors at either 6, 15 or 21 cm distances from the “anode top”; (b_6,15,21_) images of helium ion angular distributions on half of a MSPC wall detector simultaneously exposed with a circular MSPC detector at a known distance; (c_6,15,21)_ images of helium ion distributions on circular and wall MSPC detectors when assembled together forming 3D ion distributions in PFD space by applying a SketchUp Software in a FaceStyle (shaded with shadows) mode at either 6, 15 or 21 cm distances; and (d_6,15,21_) images of ion emission angular distributions of circular and wall MSPC detectors when assembled forming 3D ion distributions in PFD space by applying a SketchUp Software in a FaceStyle (X-ray) mode at either 6, 15 or 21 cm distance.

For each circular MSPC detector exposed at either 6, 15, or 21 cm distances from the “anode top”, a rectangular MSPC detector (38 cm × 81 cm) was also placed and exposed on the PFD wall simultaneously. Half of this rectangular MSPC detector (38 cm × 40.5 cm) was processed at a time in a rectangular MS-ECE chamber. The ion distribution images formed can be easily observed by the unaided eyes or photographed by a digital camera or scanned by a scanner. By combining the images of ion emission distributions on a circular MSPC detector and that of a respective rectangular MSPC wall detector, when they were simultaneously exposed, 3D images of ion distributions in PFD space (except the ground PFD base) have been constructed by applying a SketchUp Software (Fig. 4a,b,c,d). It should be noted that the white areas on all the images are high-density helium ion tracks; the whiter the area, the denser the ion fluence. This means that the white areas on the images of 6, 15 and 21 cm distances are all dense ion tracks. The dark areas on the images like those observed on the “ICSs” have registered no or negligible ion tracks. One can directly observe and diagnose at a glance the status of ion emission angular distribution image at each position on a MSPC detector corresponding to a position in the PFD space. This capability confirms the power of the MSPC image detection systems with high position-sensitivity and spatial resolution which are of importance for detailed ion emission angular distribution studies and for routine efficient applications of PFDs in medicine, science and technology.

As shown in Fig. 3 and Fig. 4(a,b,c,d), ion emission angular distributions on both circular and rectangular MSPC detectors depend on the distance of its respective circular detector from the “anode top”. By an analysis of the ion distribution images, some preliminary conclusions are made as follow:

- As can be seen in Fig. 2 and Fig. 4a6,15,21, an ion angular distribution image on each circular MSPC detector exposed at a known distance from the “anode top” is quite different from those of others. In fact, at 6, 9 and 12 cm distances of the circular MSPC detector from the “anode top”, ion tracks first appear as a bunch in the middle of the circular detector during the ECE processing followed by appearance of more ion tracks as a ring or a tube around the circular detector, as also observed directly in the transparent chambers during the MS-ECE processing by the unaided eyes. This observation implies that the ion bunches sputtered in the middle have a lower energy than those of the surrounding ion tracks on the ring. On the other hand, ion tracks on circular MSPC detectors exposed at 15, 16, 18 and 21 cm distances first appear as a ring or tube around the circular detector with gradual appearance of more ion tracks towards the center. This observation implies that for these larger distances of the circular detector from the “anode top”, ions exposing around the circular MSPC detector are of much lower energy than those developed in the middle at a later stage. As regards to rectangular MSPC detectors exposed on the PFD wall simultaneously with a circular detector, the ions appear on top of the detector and gradually fill the spaces over and between the “ICSs” which a gradual appearance of the tracks to the bottom of the detector. This observation also implies that the ions appearing on top of the wall MSPC detector have a lower energy than those appearing at a later stage. We have made similar observations with hydrogen ions which will be reported soon.
- As discussed above, the ion tracks appearing on a rectangular MSPC wall detector depend on the distance of a circular MSPC detector from the “anode top”. As shown in Fig. 4b6,15,21, ion tracks are only observed on the area of the wall detector below the level or distance of a circular MSPC detector from the “anode top”; i.e. the circular detector being the same size of the PFD cylinder base have prevented ions to be distributed to upper PFD level. This observation means that on any wall detector (Fig. 4b6,15,21), ions furnish only the area of the detector below the level or distance of its respective circular detector from the “anode top”. However, the areas on the “ICSs” formed on the wall detectors have no ion tracks since the ions have been shadowed by the cathodes. The formation of “ICSs” on the wall and cathode detectors is a new phenomenon discovered, as is discussed below.
- In order to verify if any helium ions reach the PFD cylinder ground base and to identify the source (s) of such ions, the small PC detectors (3 cm × 3 cm) laid on the ground base were processed, counted and analyzed. For small PC detectors laid between the anode and cathodes, no successful pinch was formed thus no ions were detected. However, those small PC detectors laid on the ground areas between the cathodes and PFD wall have received ions under two conditions depending on the arrangements of the small PC detectors: (1) when they were exposed to ions from the “anode top” and also from the “anode cathodes array” simultaneously with a fluence of ~3.1 × 10^5^ ions.cm^−2^ and (2) when they were exposed only to ions from the “anode top” with a lower fluence of ~1.3 × 10^5^ ions.cm^−2^, when the “anode cathodes array” ions were shielded. The difference of ion fluence of the two detector groups; i.e. ~1.8 × 10^5^ ions.cm^−2^, is therefore due to ions originated from the “anode cathodes array”, exposing also the ground base of the PFD to ions. This observation is being further verified.
- Another phenomenon observed on the wall MSPC detector is formation of an intense ion belt with a relatively higher ion track density than those of the surrounding areas (Fig. 4b21,c21,d21, in particular b_21_). This ion belt has been distinctly observed on the wall MSPC detectors during the MS-ECE processing or sometimes after the ECE process is completed. By a close analysis of the images, it seems that an overlapping phenomenon of ions emitted from the “anode top” in 4π directions and from “anode cathodes array” as radial run-away ions at a wide angle are responsible for this observation. While this observation seems to be a support for existence of two types of ion sources, as is discussed below, studies are underway to further verify this ion belt formation region.

### Discovery of two sources of ions in PFD space

**Figure 5:**
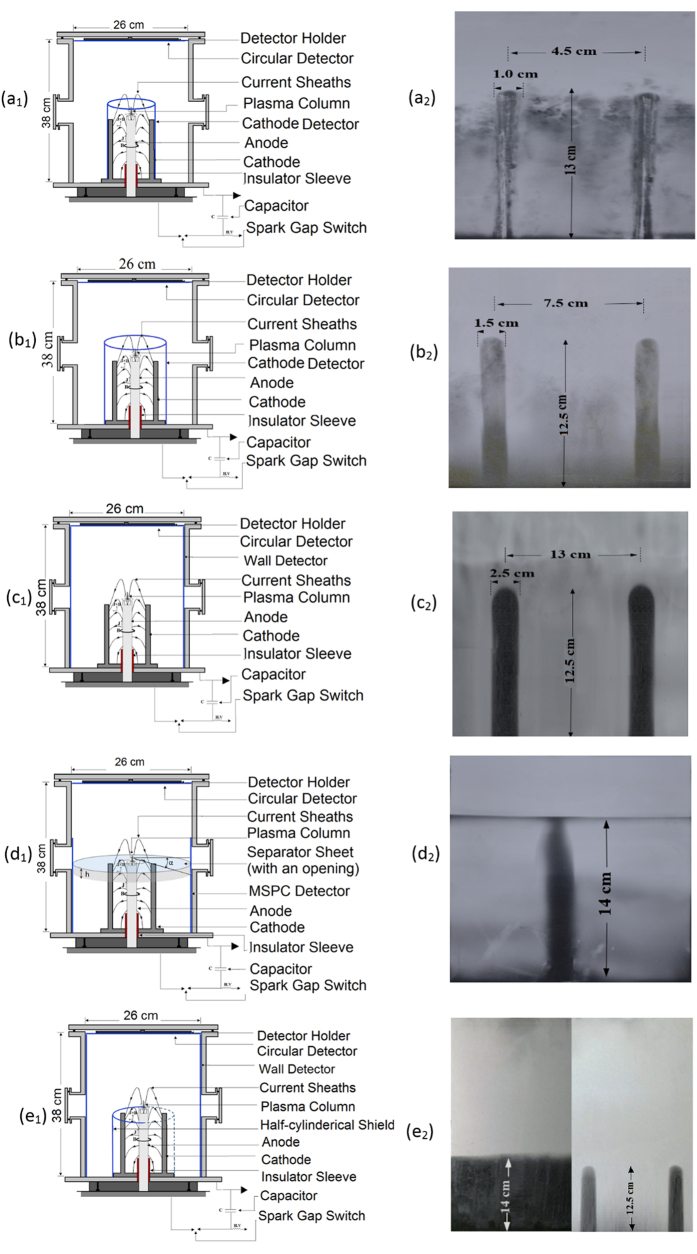
(**a–e**) Five MSPC detector set-ups and “ICS” images on detectors. (a_1,2_) A rectangular cathode MPC detector attached to the cathodes (a_1_) and images of two “ICSs” on it (a_2_); (b_1,2_) A rectangular cathode MSPC detector installed 3 cm away from the cathodes (b_1_) and image of two “ICSs” on it (b_2_); (c_1,2_) A rectangular MSPC detector placed on the PFD wall (c_1_) and images of two “ICSs” on it (c_2_); (d_1,2_) A circular sheet, 1 mm thick, with 5 cm circular opening in the middle which can separate the PFD volume into two upper and lower spaces (d_1_) and an image of one “ICS” formed on it (d_2_); and (e_1,2_) A rectangular PC sheet attached around 3 cathodes to shield ions to reach a wall detector while the other 3 cathodes were allowed to emit ions to reach another wall MSPC detector (e_1_), and two separate images; one image on the wall detector with the lower part shielded from ions emitted from the “anode cathodes array” (left) and one image of “ICS” on a wall detector with no shield in front of it (right) (e_2_).

In addition to the belt region discussed above in support of the two types of ion sources, the “ICSs” formed sharply on the wall MSPC detectors can also further verify formation of two ion sources. The “ICSs” have a length equal or near that of a cathode and a width geometrically proportionate to the cathode diameter depending on the distance of the detector from the anode. At the first glance, there was an impression that shadows are caused only by ion emission from the “anode top” in the PFD space. At the same time, the possibility that the “anode electrodes array” might play a role in formation of such shadows could not be overruled. In search to find possible sources of such shadows, 5 experiments were set-ups based on using the cathode and wall MSPC detectors at different distances from the cathodes each under a separate single pinch shot. Schematic diagrams of locations of the cathode or wall MSPC detectors in 5 experimental set-ups in the PFD space and “ICS” images formed on them are shown in Fig. 5(a,b,c,d,e).

By analysis of the ion emission images of Fig. 5(a,b,c,d,e), the role of the “anode top” and the “anode cathodes array” as two ion sources forming the “ICSs” on the cathode and wall MSPC detectors can be verified as follow:

- Figure 5(a1,a2) shows the PFD space with a rectangular cathode MSPC detector (21 cm × 51 cm) wrapped around the cathodes at 4.5 cm distance from the anode center (a_1_) and ion emission image with two “ICSs” formed on part of a rectangular detector (a_2_). This cathode detector has registered ions emitted from the “anode top” in 4π PFD space (except those ions shadowed by the anode head with 2 cm diameter). The cathode detector also registered the ions escaped radially from the “anode cathodes array” which contribute intensively to the formation of the “ICSs”. The “ICSs” formed have ~13 cm length (same as cathode length) and ~1 cm width (the same width as that of a cathode) on a dense ion track density. At 7.5 mbar pressure, only shadows of upper portion of the cathodes were registered. However, at a pressure of 20 mbar, the “ICSs” are fully developed from top to the bottom (a_2_). The gas pressure is instrumental in forming full-size “ICS” images under a tight “anode cathodes array” space. It is interesting to note that ions have also been generated in the gap between the insulator sleeve (48 mm) and the cathodes (Fig. 5a2,b2,c2,d2,e2).
- Figure 5(b1,b2) shows the PFD space with a rectangular MSPC detector (21 cm × 51 cm) installed around the cathodes’ base 3.0 cm and 7.5 cm away from the center of cathodes and the anode respectively (b_1_) and image of two “ICSs” formed on a portion of a cathode detector (b_2_). Considering such a detector arrangement, the cathode detector faces intense ions from the “anode top” and also radially from the “anode cathodes array” even at 7.5 mbar. The “ICSs” are sharp and distinct with ~12.5 cm length, the same as those of other “ICSs” and the cathodes, but with a ~1.5 cm width.
- Figure 5(c1,c2) shows the PFD space with a rectangular MSPC detector (38 cm × 81 cm) installed around the wall (c_1_), as discussed above, and image of two “ICSs” formed sharply on the detector (c_2_). The “ICSs” are sharp and distinct with a ~12.5 cm height the same as the other “ICSs” but with a ~2.5 cm width proportionate in respect to the distances to the anode center. Under this detector set-up, the “ICSs” formed have good ion contributions from the two ion sources; i.e. from the “anode top” and the “anode cathodes array”.
- Figure 5(d1,d2) shows the PFD space separated into two upper and lower spaces by a 1 mm thick circular PC sheet (~26 cm in diameter) with a circular opening (5 cm diameter) in the middle laid over the 6 cathodes (~3 mm below the “anode top” level) (d_1_), and image of one “ICS” on a smaller wall MSPC detector facing only one cathode (d_2_). This separator sheet allows the ions from the “anode top” be distributed freely in the upper PFD space forming a dense ion track region on the wall detector (d_2_ left). However, the separator sheet geometrically allows the “anode top” ions to reach the lower PFD space only though the circular opening within a ~79° cone angle formed around the z-axis; i.e. no ions through an angle α (~11°) formed with the separator line can reach the wall detector. It is geometrically expected that an ion-free band of a ~2.1 cm height is formed around the PFD wall with no ions to be registered (6d_1_). However, unlike this geometrical restriction some interesting observations are made on the wall detector (6d_2_): (i) the ion-free band is densely filled with helium ion tracks except above the “ICS”, (ii) The “ICS” formed is ~14 cm long about ~1.5 cm longer than the other “ICSs” touching the separator line which seem to be due to shadowing effect by a cathode, and (iii) the “ICS” has no sharp round top like the others. These observations confirm that the ions radially escaped from the “anode cathodes array” are responsible for filling the ion-free band with ion tracks. It may also be concluded that the “anode top” might be responsible for forming the top round shape of the “ICS”, as we have also observed in some other MSPC detectors processed.
- Figure 5(e1,e2) shows the PFD space with a rectangular PC sheet (same length as anode height), attached to only 3 cathodes in order to shield the ions from reaching the wall detector while the other 3 cathodes have no cover sheet so that the wall detector can receive radial run-away ions (e_1_), and two images from the two parts of respective wall detectors (e_2_). The image of ions (e_2_ left) has a line separating the area above the anode level with dense tracks of ions from the “anode top” and the area below the anode level with no ion tracks since the wall detector was shielded, and the image of two “ICSs” (e_2_ right) on the dense tracks of ions from the “anode top” as well as from those escaped radially.

The above experimental verifications clearly show that the ions from the “anode top” are emitted upwards, downwards and in all directions as 4π ion emission in PFD space after plasma pinch instability, as if it is a “PIS”. Ions also escape radially from the “anode cathodes array” during axial acceleration of plasma sheaths before the radial phase, as if the anode is a “LIS”. These two ion sources seem to be responsible for formation of “ICS” on the cathode and wall rectangular MSPC detectors. The above set-ups are being further studied for analysis and verification of the phenomena observed by other ions.

## Discussion and Conclusion

Emission of ions in a PFD space in conic geometry within a small solid angle has been so far confirmed with a current scientific understanding by measuring ion characteristics only at certain locations above the vacuum chamber by applying several pinch shots. Some previous studies on ion angular distributions on X or Y axes in PFDs have reported maxima or minima on or around the z-axis on angular distribution profiles^22^,^23^,^24^,^25^,^26^,^27^,^28^,^29^,^30^,^31^, or experienced wider but limited ion emission angular distribution profiles^7^,^18^,^35^,^36^,^37^,^38^. In this context, some theoretical models have also been proposed on angular distributions to verify the observations made^22^,^23^,^24^,^29^,^30^,^32^,^33^,^34^,^35^. The rationale for such contradicting results and understanding of ion emission mechanisms in a PFD space seems to be availability of only few types of detectors with limitations for real wide-angle ion emission studies in 4π PFD space.

**Figure 6:**
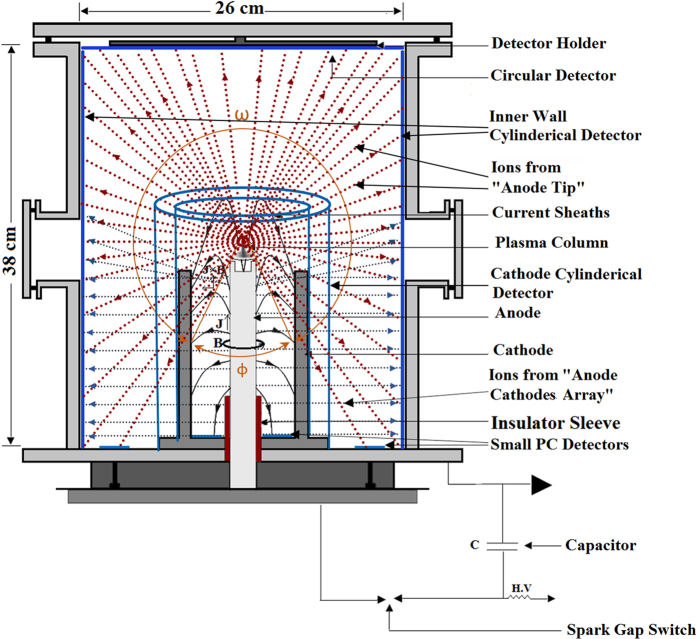
Schematic diagram of PFD space with PC detector arrangements. Schematic diagram of the inner space of the PFD showing a circular MSPC detector attached to top cylinder base facing the “anode top”; a rectangular MSPC detector laid on the inner wall; smaller rectangular MSPC detectors (one wrapped around and attached to the cathodes and another at a distance away from the cathodes, but exposed one at a time); as well as small PC detectors laid on the ground cylinder base (some on the area between the anode and cathodes as well as some on the area between the cathodes and the wall). It should be noted that each of the cathode or wall detectors was independently exposed either with a circular detector and/or small detectors on the ground PFD base.

The breakthrough and discoveries made in this study resolve the shortcomings and limitations of presently available detectors and reveal facts towards better understanding of ion emission and angular distributions in 4π PFD space. The progress made in this study include: (1) invention of position-sensitive MSPC ion image detection and MS-ECE chamber processing systems, (2) discovery of some phenomena which prove ions not only are emitted from the “anode top” upwards, downwards and almost in all directions in a 4π ion emission in PFD space after plasma pinch instability, but also intense ions are escaped radially from the “anode cathodes array” during the axial acceleration of the plasma sheath before the radial phase, and (3) discovery of formation of the “ICS” images on rectangular cathode and wall MSPC detectors. These “ICS” images seem be formed by two ion emission source mechanisms as discussed above. Based on the “experiences gained and lessons learned” during such extensive studies, some scientific facts discovered in a PFD space are further discussed and some conclusions are made as follow:

- The position-sensitive MSPC image detection systems developed in this study are unique, efficient, flexible, and practical for 4π ion emission angular distribution studies in particular in a PFD space. The detectors have desirable characteristics such as: availability in large sheets in different thicknesses; masked on both sides to prevent scratches; unique uniformity; flexibility to match with any geometry condition; high efficiency; possibility to use small (e.g. 3 cm × 3 cm) to mega-size (e.g. 38 cm × 81 cm, the size of PFD inner wall as currently constructed) detectors; transparent MS-ECE chambers with possibility to observe ion track image as gradually appear during the ECE process; possibility for direct analysis of ion angular distributions at a glance by the unaided eyes; position-sensitivity with high spatial-resolution; insensitivity to large doses of x, γ and β rays as well as non-ionizing radiations; insensitivity to ambient environmental conditions; need to a single pinch shot to obtain ion fluence and energy distribution matrix in 4π PFD space; statistical consistency for event matching; simple record keeping; need to simple home-made equipment; low cost and last but not least need to minimal training.
- These wide-angle ion image angular distribution studies show that the “anode top” emits ions in a 4π ion emission in PFD space as if it is an independent “PIS”. This 4π ion emission however is inherently restricted by the anode head diameter (e.g. 2 cm in our case) which shadows ions to reach the PFD ground within a small angle (~60^o^). This angle depends strongly on the anode diameter. Therefore, ions emitted within an angle <300^o^ with respect to the “anode top” are distributed in a 4π PFD space. Some observations made on the MSPC detectors include the “ICSs” on the cathode and wall detectors (Fig. 6a,b,c,d,e), formation of an intense ion track belt on the wall detectors (Fig. 4b21,c21,d21), formation of different ion emission images on the circular detectors placed over the “anode top” such as ring-type, ring-type with sputtered ions in the middle and near full ion formation image (Fig. 2), as well as relatively lower ion intensities on the PFD ground base. Similar observations were also made with other gases which are under investigation to be reported.
- The 4π ion emission from the “anode top” as a “PIS” and from radial run-away escape of ions from the “anode electrodes array” as a “LIS” invite proposing a new model to be called “Two-sources Ion Emission Model” which of course needs to be further investigated, developed and theorized.
- A circular MSPC detector with an ion image has infinite diameters or axes each of which can be used as a PFD axis leading to infinite 2D angular distribution profiles. A 2D ion angular distribution profile on a circular detector (Figs 3 and 4a) is one of infinite ion flux angular distributions to be plotted on X or Y axes of the PFD. Therefore each of the 2D ion angular distribution profiles reported within small solid angles using single or array of few detectors^22^,^23^,^24^,^25^,^26^,^27^,^28^,^29^,^30^,^31^, can be considered as one special case of a 2D angular distribution in a circular detector. Based on continuous ion emission angular distribution image detection on the circular detectors (Fig. 3), it can be said that the ion angular distributions in the X or Y axes strongly depend on parameters such as type and size of a detector; location of the detector in a PFD in particular its distance from the “anode top”; type of PFD and its operating conditions; type of gas and pressure; number of pinch shots and pinch strength; statistical variations from shot to shot; and expertise in ion detection and measurements. Therefore, small detectors commonly applied in such studies will not provide continuity of the distributions even if large number of such small detectors are used. Therefore, some previous observations on angular distributions and models proposed have been more based on the detectors used and the location of the detector on or around the z-axis.
- Helium ion energies were determined to be from ~0.3 to ~4.5 MeV by applying a “paired-detector comparison method” using PC detector templates calibrated to alpha particles of known fluence and energy, as discussed above. For ions such as nitrogen, neon, argon, or other heavier gases, the energy detection range of the PC detector is extended to much higher energies since PC detectors register heavier ions of higher stopping power with a higher efficiency over a broad energy range, provided that the detectors are calibrated to identical ions of known fluence and energy.
- The formation of wide-angle ion fluence matrix information with position sensitivity on a processed MSPC detector under a single pinch shot, with capacity to be directly observed and diagnosed by the unaided eyes, are assets in promoting efficient applications of PFDs such as radioisotope production, thin film material processing, synthesis of nanophase materials, ion implantation, lithography, radiography imaging and neutron sources^1^,^2^,^3^,^4^,^5^. In fact the ion fluence at any desired position in the PFD space can be directly observed, analyzed and diagnosed on MSPC detectors at a glance by the unaided eyes. Therefore, each single position or location in PFD space can be utilized efficiently for exposing specimen (s) to a known ion fluence and energy so that thousands of specimens might be exposed at a time under a single shot or more shots if needed to have higher ion fluence. By “lessons learned and experiences gained” in our studies, almost all locations in a 4π space of a PFD depending on its type, size and energy can be made available to be used as a strong ion source with enhanced efficiency for wide-spread large-scale applications in medicine, science and technology; what have been very minor and limited so far.
- The position-sensitive MSPC ion image detection systems invented and successfully applied in this study clearly demonstrate that the PFD ion emission beams are more reproducible than what is presently observed based on 2D ion angular distributions on X or Y axes, a fact we have clearly observed. In fact this is due to the power of the MSPC image detection systems invented that require only a single shot to register all ions under a single shot what makes such a claim more feasible.

More studies have been or being further carried out in our laboratory for hydrogen, deuterium, helium, nitrogen, neon and other ions in support of what have been reported in this paper. The MSPC image detection systems applied, innovations and discoveries made on the 4π ion emission distributions in PFD space are believed to open new horizons for advanced experimental and theoretical studies to better understand ion emission mechanisms, to develop new theoretical models and to promote efficient advanced applications of PFDs in medicine, science and technology.

## Methods

### Amirkabir 3.5 kJ Plasma Focus Device

The Amirkabir plasma focus device (PFD), as used in this study, is a Mather-type 3.5 kJ device^49^. A Mather-type device is a compact device with two concentric cylindrical electrodes enclosed in a vacuum chamber. In order to operate the device, the chamber is first evacuated to a low pressure in the order of 10^−5^ mbar or lower and then it is filled with a known gas such as hydrogen, deuterium, helium, nitrogen, oxygen, etc. at a known pressure of several mbar.

The PFD used in this study is an upright cylinder with 26 cm inner cylinder base diameter, 38 cm inner height and 81 cm inner circumference (Fig. 6). It has an “anode cathodes array” assembly at the center which consists of a central hollow copper cylindrical anode (13.3 cm long with 2.0 cm diameter) and six copper cathodes (13 cm long with 0.9 cm diameter) (Fig. 3) placed on a 1 cm thick copper base. A Pyrex glass tube with an effective length of 48 mm acts as the insulator sleeve. In the studies performed, the chamber vacuum was maintained at about 10^−5^ mbar. A Rogowski coil was used to record the current signal. The device was charged by a 40 μF capacitor up to 12 kV. Total external inductance of the device is 115 nH. For exposing the MSPC detectors in the PFD, the gas pressure was maintained at 7.5 mb for helium gas. However, in an experimental set-up when a rectangular MSPC detector was wrapped around and attached to the cathodes, a higher pressure of 20 mbar was required to trigger a successful pinch. In this study, helium was the main gas but other gases like hydrogen, deuterium, nitrogen and neon gases were also used to verify near 4π ion emission observations and formation of “ICS” on the MSPC detectors which will be reported soon.

Figure 6 shows schematic diagram of the PFD with also circular and rectangular MSPC detectors as well as small PC detectors placed at specified locations in the PFD ground base; ions emitted and distributed from the “anode top” upwards, downwards and in all directions in 4π PFD space; and ions escaped radially from the “anode cathodes array”. Other special detector arrangements have also been made, as discussed under each experimental set-up as discussed above (Fig. 5a,b,c,d,e).

### Ion image detection system; principles and mechanisms

When a heavy charged particle like a helium, nitrogen or neon ion impinges on a polymer track detector like polycarbonate (PC), it breaks the polymer bonds into free radicals along its trajectory forming a latent track. The latent track can be easily enlarged on such polymer detectors by chemical etching^50^, or by an ECE method under optimized conditions to a point observable by the unaided eye^46^,^47^,^48^,^51^,^52^,^53^,^54^,^55^. In this study, PC detectors of different sizes in particular as MSPC detectors were applied and processed in MS-ECE chambers by applying the 50 Hz-HV ECE method^46^,^47^,^48^. The ion track image MSPC detection and ECE processing systems consist of several parts and steps including: ion image PC detector (s), detector exposure to heavy ions in a PFD, MS-ECE chamber system, MS-ECE processing conditions, 50 Hz - HV generating system, and ion fluence and energy distribution determination.

### Ion image MSPC detectors

The ion track image detectors as used in this study is basically PCTDs of a desired size and thickness depending on the application; i.e. from a small size to a mega-size detector, as applied for the first time in PFDs in these studies. An ion image MSPC detector is basically a piece of PC sheet cut from a larger PC sheet of 250, 500, 750 or 1000 μm thicknesses (masked on both sides to prevent scratches) which are commercially available in any common plastic market at a low cost. The PCTDs used in most of these studies were 250 μm thick. They were applied either as circular MSPC detectors (26 cm diameter = 531 cm^2^ area, the same size as that of the PFD cylinder base) or rectangular MSPC detectors (38 cm × 81 cm = 3078 cm^2^, almost equal to the PFD cylinder inner wall area) bent around the PFD cylinder wall to make a wall detector; rectangular MSPC cathode detectors with two different sizes; 21 cm × 31 cm (when tightly wrapped around the cathodes with 4.5 cm distance from the anode center) and 21 cm × 51 cm (when placed at 3 cm distance from the cathodes with a 7.5 cm distance from the anode center) as “cathode detectors”. Small size 3 cm × 3 cm PC detectors were laid on the PFD ground base areas between the anode and cathodes and between the cathodes and PFD wall. Theses PC detectors are also shown in Fig. 5(a,b,c,d,e) and Fig. 6 as installed in the PFD space.

### Detector exposure to heavy ions in a PFD

Six circular MSPC detectors were exposed each at a time at either 6, 9, 12, 15, 18 or 21 cm distances from the “anode top” to helium ions at a single pinch shot, as shown in Figs 2 and 6. The time interval between different shots was usually one hour or occasionally 24 hours if the system or time did not allow to continue. However, since PC detectors have rather nil fading of induced ion tracks, this time interval causes no fading of induced tracks even at much longer time intervals. Each rectangular wall MSPC detector was exposed simultaneously with a circular detector when placed at either 6, 15 or 21 cm distances from the “anode top”. Small detectors were also laid on the PFD ground base simultaneous to other MSPC detector exposures. By such arrangements, ions emitted and distributed in the PFD space at a single shot have been registered by all the detectors installed in the PFD space as shown in Fig. 6 above. The MSPC detectors are position-sensitive and can simply show the level of ion density at any point in a PFD space after adequate MS-ECE processing, even by the unaided eyes.

### MS-ECE chamber processing system

**Figure 7:**
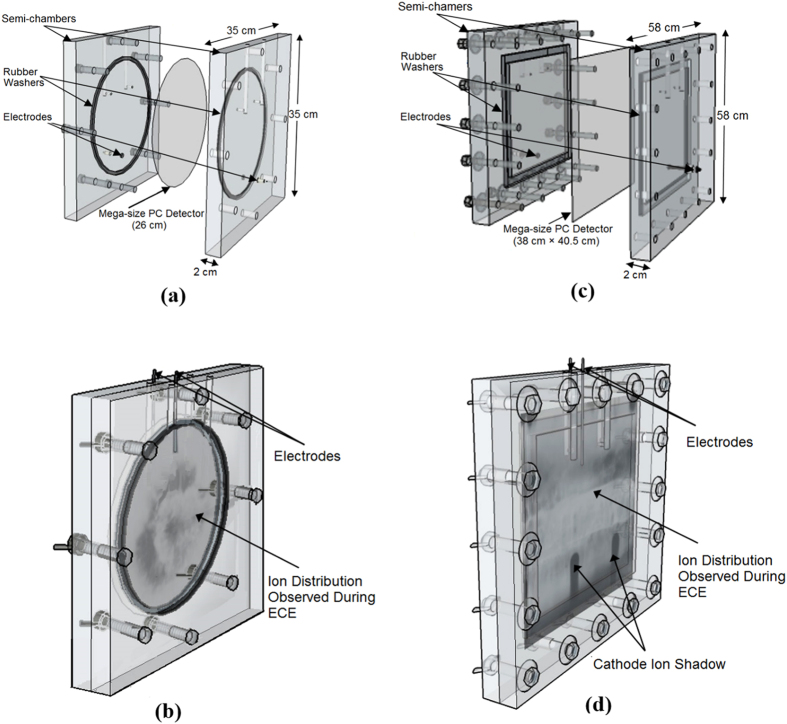
(**a–d**) Schematic diagrams of two designs of MS-ECE chambers. MS-ECE chambers; (**a**) a circular MS-ECE chamber with a circular MSPC detector in the middle when the chamber components and the detector are not assembled and (**b**) when they have been assembled showing also a helium ion angular distribution image on the circular MSPC detector as observed during ECE; as well as (**c**) a rectangular MSEC chamber with a rectangular MSPC detector in the middle when chamber components and the detector are not assembled and (**d**) when they have been assembled showing a helium ion distribution image as observed during the ECE processing. The images of ion emission angular distributions on both MSPC detectors in particular those of the “ICSs” are distinctly observable by the unaided eyes during and in particular after the end of an optimized ECE processing.

A MS-ECE chamber system consists of two equal MS transparent Plexiglas semi-chambers which can be assembled together by a number of bolts and wing nuts. A MSPC detector can be held tight in place between the two semi-chambers by two large rubber washers to insulate the two-semi-chambers from each other. The semi-chambers are filled with an optimized etchant. A high voltage is applied across the two semi-chambers through two stainless steel electrodes by a 50 Hz-HV generator. The design characteristics of single or multi-chamber ECE systems and their operation have been well documented in the literature^46^,^47^,^48^,^52^,^53^,^56^. For this study, MS-ECE chambers were constructed using Plexiglas (2 cm thick) with 4 different designs; a circular MS-ECE chamber for processing 26-cm circular MSPC detectors (24.5 cm effective etched diameter), a rectangular MS-ECE chamber to process a 38 cm × 41.5 cm rectangular detector (34 cm × 34 cm effective etched area^57^ (i.e. half of a ~38 cm × 81 cm wall detector), and two smaller MS-ECE rectangular chambers to process relatively smaller cathode MSPC detectors, as discussed in section (a) above. Figure 7(a,b,c,d) shows schematic diagrams of two different designs of MS-ECE chambers; a circular MS-ECE chamber, when chamber components and the detector are not assembled (a) and when they have been assembled showing also a helium ion angular distribution image on the circular MSPC detector as observed during ECE processing (b); as well as a rectangular MS-ECE chamber; when the chamber components and the detector are not assembled (c), and when they have been assembled showing also a helium ion distribution image on the rectangular MSPC detector as observed during the ECE processing showing also the “ICSs” (d). The images of ion emission angular distributions on both MSPC detectors in particular those of the “ICSs” are distinctly observable by the unaided eyes during and in particular after the end of an optimized ECE processing. The MS-ECE chamber system discussed is a new powerful method allowing also direct observation of ion images developed on thee MSPC detectors at any stage of the ECE processing by the unaided eyes. The small 3 cm × 3 cm PC detectors laid on the ground PFD base were processed in triplet ECE multi-chamber system designed and commonly used in our laboratory^56^.

### High voltage generating system

The MS-ECE processing method as used in this study requires a 50 Hz - HV generator to provide adequate field strength across the MS-ECE chamber which holds a MSPC detector tight in place between two semi-chambers. Home-made 50 Hz - HV generators for provision of an adequate field strength across the MSPC detectors have been designed, constructed and used in our studies^46^,^47^,^48^. The generator upgrades the 50 Hz–220 V output of an electricity main by an autotransformer and a step-up transformer to a desired voltage. An optimized field strength at 50 Hz frequency of minimum 32 kV.cm^−1^ is applied through two stainless steel electrodes inserted in the two semi-chambers (filled with an etchant) holding a MSPC detector tight in place for ECE processing. The high voltage across the chamber is checked for constancy of the high voltage applied before and during the processing.

### MS-ECE processing etchant solution

The MSPC detectors (250 μm thick) were processed by applying 50 Hz–2 kV field strength to a relevant MS-ECE chamber filled with an optimized etchant solution. The etchant is a mixture of 15 g KOH + 40 g C_2_H_5_OH + 45 g H_2_O (PEW solution) at 26 ± 1 °C for a duration of usually 3 hours or sometimes longer to better observe an ion emission image when required. It is interesting to note that the ion emission images in particular with the “ICSs” on it can be observed even after a short period of 30 to 60 minutes of ECE processing. When the MS-ECE processing is complete, the images fully formed can be observed, diagnosed and analyzed by the unaided eyes or by microscopic counting and analysis.

### Ion fluence and energy distribution determination

For quantitative ion fluence distribution diagnosis and analysis, ion track density and track diameters were determined under a light microscope. At high track densities, accurate determination of track densities and diameters and in turn ion energy becomes rather difficult and inaccurate by normal microscope counting. Therefore, in order to better quantify track densities and diameters (and in turn ion energy) in particular at high track densities, a new “paired-detector comparative method” recently developed in our laboratory was applied which will be reported soon. In this comparative method, a microscopic field image of helium ion tracks of a position or location of a MSPC detector exposed to helium ions with unknown fluence and energy in the PFD is compared with a microscopic image of alpha tracks of known fluence and energy in PC detectors produced as templates for this purpose. A number of PC detectors (used as templates) has been calibrated in terms of alpha fluence up to ~6.0 × 10^6^ alphas.cm^−2^ and alpha energy from ~0.3 to ~5.0 MeV by using a well collimated ^241^Am alpha source in air at room temperature.

## Additional Information

**How to cite this article**: Sohrabi, M. *et al*. Breakthrough in 4π ion emission mechanism understanding in plasma focus devices. *Sci. Rep.* **6**, 38843; doi: 10.1038/srep38843 (2016).

**Publisher's note:** Springer Nature remains neutral with regard to jurisdictional claims in published maps and institutional affiliations.
